# A Coding Variant in the Gene Bardet-Biedl Syndrome 4 (*BBS4*) Is Associated with a Novel Form of Canine Progressive Retinal Atrophy

**DOI:** 10.1534/g3.117.043109

**Published:** 2017-05-22

**Authors:** Tracy Chew, Bianca Haase, Roslyn Bathgate, Cali E. Willet, Maria K. Kaukonen, Lisa J. Mascord, Hannes T. Lohi, Claire M. Wade

**Affiliations:** * School of Life and Environmental Sciences, Faculty of Science, University of Sydney, 2006, Australia; † Sydney School of Veterinary Science, Faculty of Science, University of Sydney, 2006, Australia; ‡ Sydney Informatics Hub, Core Research Facilities, University of Sydney, 2006, Australia; § Department of Veterinary Biosciences, University of Helsinki, 00014, Finland; ** Research Programs Unit, Molecular Neurology, University of Helsinki, 00014, Finland; †† Folkhälsan Institute of Genetics, University of Helsinki, 00014, Finland

**Keywords:** Hungarian Puli, whole-genome sequencing, blindness, obesity, infertility

## Abstract

Progressive retinal atrophy is a common cause of blindness in the dog and affects >100 breeds. It is characterized by gradual vision loss that occurs due to the degeneration of photoreceptor cells in the retina. Similar to the human counterpart retinitis pigmentosa, the canine disorder is clinically and genetically heterogeneous and the underlying cause remains unknown for many cases. We use a positional candidate gene approach to identify putative variants in the Hungarian Puli breed using genotyping data of 14 family-based samples (CanineHD BeadChip array, Illumina) and whole-genome sequencing data of two proband and two parental samples (Illumina HiSeq 2000). A single nonsense SNP in exon 2 of *BBS4* (c.58A > T, p.Lys20*) was identified following filtering of high quality variants. This allele is highly associated (*P_CHISQ_* = 3.425*e*^−14^, *n* = 103) and segregates perfectly with progressive retinal atrophy in the Hungarian Puli. In humans, *BBS4* is known to cause Bardet–Biedl syndrome which includes a retinitis pigmentosa phenotype. From the observed coding change we expect that no functional BBS4 can be produced in the affected dogs. We identified canine phenotypes comparable with *Bbs4*-null mice including obesity and spermatozoa flagella defects. Knockout mice fail to form spermatozoa flagella. In the affected Hungarian Puli spermatozoa flagella are present, however a large proportion of sperm are morphologically abnormal and <5% are motile. This suggests that BBS4 contributes to flagella motility but not formation in the dog. Our results suggest a promising opportunity for studying Bardet–Biedl syndrome in a large animal model.

Progressive retinal atrophy (PRA) (OMIA #000830-9615) is the most common cause of hereditary blindness in the domestic dog (*Canis lupus familiaris*), affecting >100 pure breeds (Whitley *et al.* 1995). It is clinically and genetically heterogeneous and encompasses several forms of disease which vary by etiology, rate of progression, and age of onset (Downs *et al.* 2014a). The typical characteristics are gradual night, followed by day vision loss due to the degeneration of rod and cone photoreceptors, and this degeneration continues until the affected animal is completely blind (Parry 1953). Ophthalmic features that become apparent as the retina deteriorates include tapetal hyper-reflectivity, vascular attenuation, pigmentary changes, and atrophy of the optic nerve head (Parry 1953; Clements *et al.* 1996; Petersen-Jones 1998).

PRA is recognized as the veterinary equivalent of retinitis pigmentosa (RP) in humans due to the clinical and genetic similarities between the disorders (Petersen-Jones 1998; Cideciyan *et al.* 2005; Zangerl *et al.* 2006; Downs *et al.* 2011). RP is a common cause of blindness in humans and affects ∼1 in 4000 people (Hamel 2006). There are very limited treatment options for both PRA and RP at present (Hamel 2006). For this reason, the dog has become a valuable large animal model for retinal degeneration, in particular, for testing the efficacy of novel therapeutics such as gene therapy (Pearce-Kelling *et al.* 2001; Acland *et al.* 2001; Narfström *et al.* 2003; Cideciyan *et al.* 2005; Beltran *et al.* 2012; Pichard *et al.* 2016). As of 2016, 256 retinal disease-associated genes were identified for humans (https://sph.uth.edu/retnet/). Some of these genes cause nonsyndromic RP, while others contribute to syndromic disorders such as Bardet–Biedl syndrome (BBS) (Hamel 2006).

Currently, retinal dystrophies in 58 domestic dog breeds have been linked to at least 25 mutations in 19 different genes (Miyadera *et al.* 2012; Downs *et al.* 2014b). Canine PRA is typically inherited in an autosomal recessive pattern, although two forms that are X-linked (Vilboux *et al.* 2008) and one that has dominant inheritance have been reported (Kijas *et al.* 2002, 2003). Many of these discoveries in the canine were made using candidate gene studies, linkage mapping and genome-wide association studies (GWAS) followed with fine mapping (Acland *et al.* 1999; Goldstein *et al.* 2006; Kukekova *et al.* 2009; Downs *et al.* 2014b). This success has been facilitated by the unique breeding structure of dogs. Intense artificial selection, genetic drift, and strong founder effects have resulted in stretches of linkage disequilibrium (LD) that can persist for several Mb within breeds, but only tens of kb across breeds (Lindblad-Toh *et al.* 2005). This species population structure has allowed for the successful mapping of Mendelian traits with fewer markers and subjects compared to human gene mapping studies: as few as 10 unrelated cases and 10 controls (Karlsson *et al.* 2007; Frischknecht *et al.* 2013; Jagannathan *et al.* 2013; Willet *et al.* 2015; Gerber *et al.* 2015; Wolf *et al.* 2015). Such methods are accepted to work extremely well for mapping monogenic traits that segregate within a single breed.

Despite this achievement, there are still many forms of PRA in several breeds of dog that have yet to be genetically characterized. Traits with underlying genetic heterogeneity and a late onset are notoriously difficult to map using linkage or GWAS methods (Hirschhorn and Daly 2005; Korte and Farlow 2013). Although PRA is collectively common, individually, specific forms are relatively rare and it may take many generations until an adequately sized cohort of unrelated case samples are collected. The genetic heterogeneity of PRA can complicate the results of linkage mapping and GWAS, as different causative variants and genes can be responsible for an identical phenotype. In addition, both linkage and GWAS rely on markers to be in LD and segregate with the disease gene, making it difficult to detect rare or *de novo* variants (Hirschhorn and Daly 2005).

Since the advent of whole-genome sequencing (WGS) and whole exome sequencing technologies, the discovery of causal variants for rare or genetically heterogeneous diseases has become more rapid with fewer case samples necessary for success. One study design of note that has been used in human and more recently in canine studies is the sequencing of parent-proband trios (Zhu *et al.* 2015; Sayyab *et al.* 2016). As this method provides the chance for earlier diagnosis than previously possible, this gives patients the opportunity to access more personalized treatment options (Farwell *et al.* 2015; Zhu *et al.* 2015; Sawyer *et al.* 2016).

In a preliminary study, extensive screening of 53 genes associated with autosomal recessive PRA or RP revealed no putative variants that could be associated with PRA in the Hungarian Puli breed (Chew *et al.* 2017). Here, we combine genotyping array data and WGS data of a parent-proband trio with an additional half-sibling case to identify a potentially novel canine PRA gene. We successfully identify a highly associated mutation in exon 2 of *BBS4* (c.58A > T, *P_CHISQ_* = 3.425*e*^−14^, *n* = 103) that segregates perfectly with the disease phenotype. This mutation encodes a premature stop codon which is expected to result in complete loss of function of the BBS4 protein. The association of *BBS4* with canine PRA is a novel finding and presents the first description of an associated variant for PRA in the Hungarian Puli.

## Materials and Methods

### Samples

This study involved 255 dogs (*C. lupus familiaris*) that comprised 103 Hungarian Puli and 152 Hungarian Pumi samples. This sample cohort included 14 Hungarian Pulis segregating PRA in an autosomal recessive pattern from a previous study (Chew *et al.* 2017). Three affected Hungarian Pulis (USCF516, USCF519, and USCF1311) were diagnosed with PRA at the age of 2 yr by registered specialists in veterinary ophthalmology. Diagnosis was based on observed ophthalmologic changes including vascular attenuation, hyper-reflectivity, and reduced myelination in the optic nerve head. The parents (USCF347, USCF524, and USCF525) were similarly tested and confirmed as PRA clear. The remaining dogs were >3 yr of age and had normal vision as reported by their owners or veterinarians. Hungarian Pumis are a very closely related breed to the Hungarian Pulis and have been considered as a unique breed only since the 1920s, so were considered as a compatible cohort for this study.

Biological samples from the 255 dogs were collected either as EDTA-stabilized whole blood or buccal cells using noninvasive swabs (DNA Genotek) or indicating Whatman FTA Cards (GE Healthcare). Genomic DNA was isolated from whole blood using the illustra Nucleon BACC2 kit (GE Healthcare) or from buccal cells on swabs using the Performagene Kit. For samples collected on an FTA card, DNA on discs was purified according to the manufacturer’s guidelines.

We ensured that recommendations from the Australian Code for the Care and Use of Animals for Scientific Purposes were strictly followed throughout the study. Animal ethics approval was granted to conduct this research by the Animal Ethics Committee at the University of Sydney (approval number N00/9–2009/3/5109, September 24, 2009) and the State Provincial Office of Southern Finland (ESAVI/6054/04.10.03/2012).

### Genotyping array data

Genotyping array data of 14 Hungarian Puli and WGS data of a parent-proband trio and one additional half sibling case (USCF347, USCF516, USCF519, and USCF525) were obtained from the preliminary study (Chew *et al.* 2017). Genotyping was performed on the CanineHD BeadChip array (Illumina, San Diego, CA) by GeneSeek (Lincoln, NE). WGS was performed as 101 bp, paired-end reads on the Illumina HiSeq 2000 by the Ramaciotti Centre, University of New South Wales, Kensington. The Illumina TruSeq DNA polymerase chain reaction (PCR)-free kit was used to prepare the libraries. The four samples were barcoded and sequenced on two lanes of the sequencing machine. For additional information on sample and data collection, refer to the supplementary information in Chew *et al.* (2017). Sample information for this study can be found in Supplemental Material, File S1.

### Candidate gene selection

Comprehensive screening of 53 PRA loci in the Hungarian Puli family revealed no obvious functional variants for the phenotype of interest (Chew *et al.* 2017). To identify novel candidates, regions concordant with a recessive inheritance pattern were identified using two case (USCF516 and USCF519) and 12 control dogs that were genotyped at 172,938 SNP markers on the CanineHD array. The control dogs included three PRA-clear parents (USCF347, USCF524, and USCF525). Only markers that were genotyped as homozygous for the minor allele in cases, heterozygous in the parents, and heterozygous or homozygous for the reference allele in the remaining nine control dogs were regarded as target loci (Microsoft Excel 2010).

Candidate genes were selected from the region with the highest frequency and density of concordant SNPs. LD in purebred dogs can span several Mb long (Lindblad-Toh *et al.* 2005), thus we considered markers within 5 Mb to be in a single haplotype block. Using the corresponding syntenic positional region in the mouse reference genome (mouse genome assembly GRCm38, January 2012 build, the Genome Reference Consortium), we restricted our analysis to genes with a known phenotypic connection to vision using the Mouse Genome Browser (http://jbrowse.informatics.jax.org/). Any genes within the identified regions that were not already assessed in the preliminary PRA gene screening study (Chew *et al.* 2017) were chosen as positional candidate genes and considered for further analysis.

### Whole-genome sequence processing and putative mutation detection

Next-generation sequencing data from two cases (USCF516 and USCF519) and two parental controls (USCF347 and USCF525) were aligned to CanFam 3.1 (Hoeppner *et al.* 2014). Reads were aligned as pairs using the Burrows–Wheeler Alignment tool with default parameters (Li and Durbin 2009). PCR duplicates were marked using Picard (http://broadinstitute.github.io/picard/). Local realignment around insertion-deletions (indels) was performed using the Genome Analysis Tool Kit (GATK) (McKenna *et al.* 2010; DePristo *et al.* 2011).

High quality variants were called for all four individuals simultaneously over 12 candidate genes that were selected from the locus with the highest density of SNPs concordant with autosomal recessive inheritance. Raw variants were first called using HaplotypeCaller provided by GATK (Van der Auwera *et al.* 2013; McKenna *et al.* 2010). SNPs were then removed if Quality Depth <2.0, Fisher Strand >60.0, Mapping Quality <40.0, HaplotypeScore >13.0, MappingQualityRankSum <−12.5, and ReadPosRankSum <−8.0. Indels were removed if Quality Depth <2.0, Fisher Strand >200.00, and ReadPosRankSum <−20.0.

The remaining high quality SNPs and indels were annotated using Variant Effect Predictor provided by Ensembl (McLaren *et al.* 2010). Known population variants obtained from publically available data were not considered as candidates (Lindblad-Toh *et al.* 2005; Vaysse *et al.* 2011; Axelsson *et al.* 2013). Exonic variants were manually evaluated for genotype quality and conformation to the expected inheritance pattern using SAMtools tview (Li *et al.* 2009) and the UCSC Genome Browser. Remaining variants which were predicted by SIFT (Sim *et al.* 2012) to be deleterious (<0.05) were then considered for genotype validation and segregation analysis in the wider population by Sanger sequencing.

### Variant validation and segregation analysis

The pedigree relationships among the 14 array-genotyped individuals for which registered (Australian National Kennel Council) pedigree data were available were tested through identity-by-descent proportions calculated using PLINK (Purcell *et al.* 2007).

To confirm that the identified mutation was not a sequencing error and that the variant was concordant with the Mendelian expectation of the disorder phenotype, we genotyped 103 Hungarian Puli and 152 Hungarian Pumi for the candidate causative mutation c.58A > T in *BBS4* using PCR and Sanger sequencing.

Forward (5′-GTTAGCAAGATACATGGTGTTGC-3′) and reverse (5′-GACTATTACTGCTTTCCCCAAAA-3′) primers were designed with Primer3 (Rozen and Skaletsky 2000) to amplify a 225 bp product flanking the candidate mutation. PCR was carried out using the AmpliTaq Gold 360 Master Mix (Applied Biosystems) in a 20 µl reaction volume. Following denaturation at 95° for 15 min, samples underwent amplification for 35 cycles at 95° for 30 sec, 55° for 30 sec, 72° for 45 sec, followed by a final elongation step at 72° for 10 min. For the purification of each sample, 7 µl of PCR product was dispensed into 3 µl of master mix containing 10× shrimp alkaline phosphatase (SAP) buffer, 1 U SAP, 1 U Exo I, and water. Enzymatic activity was enabled for 30 min at 37° and was then deactivated during 15 min at 80°. Sanger sequencing of purified PCR products was carried out by the Australian Genome Research Facility at Westmead in accordance with the vendor’s instructions.

### Assessment of Bbs4^−/−^ mouse phenotypes in the dog

In addition to retinal degeneration, previous studies with *Bbs4*-null mice demonstrated that the protein is implicated in obesity and infertility caused by a failure to form spermatozoa flagella (Mykytyn *et al.* 2004; Aksanov *et al.* 2014). Veterinarians who assessed the three affected Hungarian Puli anecdotally described these individuals as obese. A fertility assessment was performed for the sole intact male (USCF519; USCF347 is female and USCF1311 was desexed) by an animal reproduction specialist at the University of Sydney. Semen characteristics were compared with previously reported data for healthy dogs as a breed-matched control was not able to be obtained (Schaer 2009). The sperm-rich fraction of semen was collected by digital stimulation into a polypropylene test tube. Semen volume and color were noted immediately. Spermatozoa were observed using phase contrast microscopy at 100× magnification, and motility was subjectively determined. An aliquot of semen was smeared onto a slide for morphology assessment under oil at 1000× magnification, using previously described criteria (Feldman and Nelson 1987). Sperm count was determined by use of a hemocytometer.

### Data availability

File S1 contains a list of accession numbers for available sample data. Genotyping array data were deposited in NCBI’s Gene Expression Omnibus under the accession number GSE87642. WGS data for four Hungarian Puli (BAM files) can be obtained from NCBI’s Sequence Read Archive under BioProject accession number PRJNA344694.

## Results

### Target loci and candidate genes

**Figure 1:**
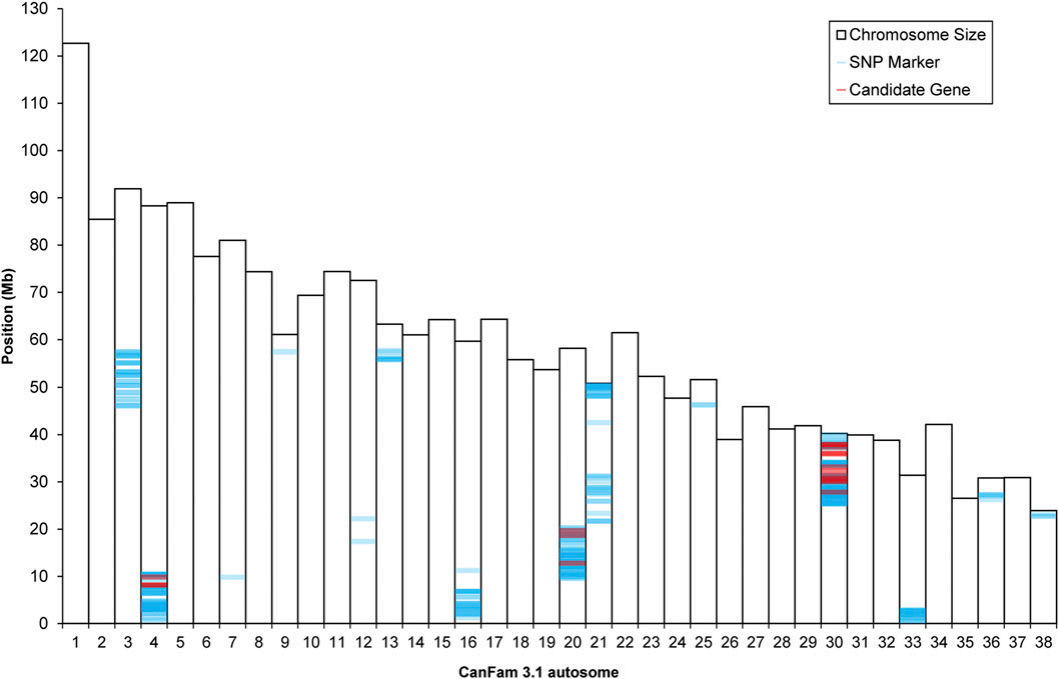
Positions of SNP array markers that segregate with the PRA phenotype and candidate genes are identified. Concordant markers are indicated in blue. Color opacity describes the density of concordant markers with darker hues corresponding with higher concordant marker density. Candidate genes are depicted in red. The locus with the highest frequency and density of markers is chr30: 25,254,123–39,976,525, with 103 markers and 12 candidate genes residing on the region. Following this is chr4: 556,510–10,473,708 with 61 markers and three candidate genes and chr20: 9,562,689 – 20,226,838 with 60 markers and three candidate genes.

Of the 172,938 SNP markers that were genotyped on the CanineHD BeadChip array for two cases and 12 controls, 363 markers segregated with PRA. Chromosome 30 (chr30) position 25.3–40.0 Mb demonstrated the highest density of concordant SNPs with 103 markers (Figure 1). This region is syntenic to mouse chromosome 9, 55.5–96.3 Mb (GRCm38/mm10 Assembly). The mouse phenome browser indicated that in this region 13 genes involved in vision have been identified, of which 12 are not currently known to be implicated in canine PRA. Chr4 position 0.5–10.5 Mb had the second most (*n* = 61) number of concordant markers. This region is syntenic to mouse chr13, 9.5–14.5 Mb, and chromosome 8, 122.7–127.7 Mb. Followed by this region is chr20 position 9.5–20.3 Mb with 60 concordant markers. This is syntenic to mouse chr6, 100.0–110.0 Mb. The mouse phenome browser revealed three candidate genes on each of the chr4 and chr20 regions. A total of 18 genes were selected as positional candidates in the current study (Table S1).

### WGS and variant detection

Sequencing on the Illumina HiSeq 2000 produced an average of 171 million raw reads per dog. Of these reads, 99.3% were successfully mapped to the CanFam 3.1 reference genome, resulting in an average mapped coverage of 6.9× per individual.

In the 18 selected candidate genes, 2726 high quality SNPs were detected, 1918 of which are not currently known population variants (Table 1; Lindblad-Toh *et al.* 2005; Vaysse *et al.* 2011; Axelsson *et al.* 2013). Of the 44 exonic SNPs, there were 27 synonymous, 16 missense, and one nonsense SNP. Two of the missense SNPs, one at *MEGF11*, chr30: 30,251,670 and the other at *STRA6*, chr30: 37,344,538, followed the expected inheritance pattern. Both were predicted by SIFT to be tolerated (*P* = 1) and therefore were not considered for further analysis. The single nonsense SNP detected occurred at *BBS4*, chr30: 36,063,748 and followed the expected inheritance pattern. This was predicted to be a deleterious mutation and was considered for validation and segregation analysis.

**Table 1:** Number of SNP and indel variants detected after applying standard hard filtering criteria

Filtering Criteria	SNP	Indel
High quality variants in candidate regions	2726	912
Not a common in canine population	1918	900
Exonic variants (total)	44	4
Synonymous	27	—
Missense	16	—
Nonsense	1	—
In-frame insertion	—	1
In-frame deletion	—	2
Multiple nucleotide polymorphism	—	1
Manual check for quality and inheritance pattern	3	0
Predicted as deleterious by SIFT (<0.05)	1	0

A total of 912 indels were detected, 900 of which are not currently known population variants (Table 1). Four of these were exonic, and by manual inspection none of these followed the expected inheritance pattern and so were not considered for further analysis.

### Validation and segregation of putative nonsense variant in BBS4

**Figure 2:**
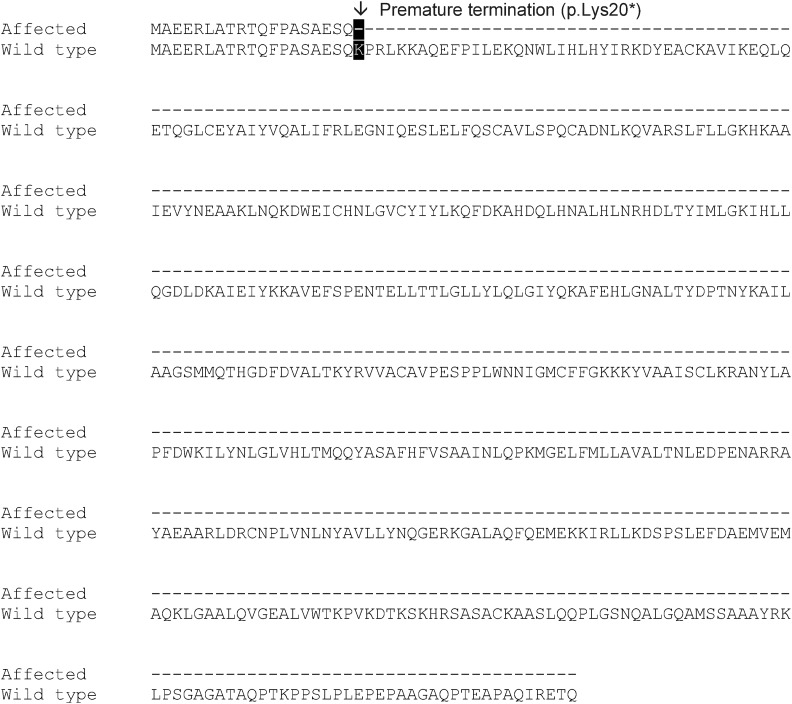
BBS4 protein sequence alignment of affected dogs containing the c.58A > T SNP and of the wild-type protein. The SNP in affected dogs results in a premature stop codon (p.Lys20*). Hyphens (-) refer to missing amino acids in the affected dogs relative to the wild-type protein.

A single, putative functional coding variant that passed all hard filtering criteria was identified. The variant results in a stop-gained mutation in *BBS4* and is predicted to be deleterious. We manually completed the annotation of *BBS4* in the CanFam 3.1 reference genome as exon 1 was evidently missing (refer to File S2 for a full description of the methods used). The complete canine BBS4 protein can be accessed through Genbank (accession KX290494). In the complete *BBS4* gene, the putative mutation results in a premature stop codon (p.Lys20*) as a result of a c.58A > T SNP in exon 2 (Figure 2).

**Figure 3:**
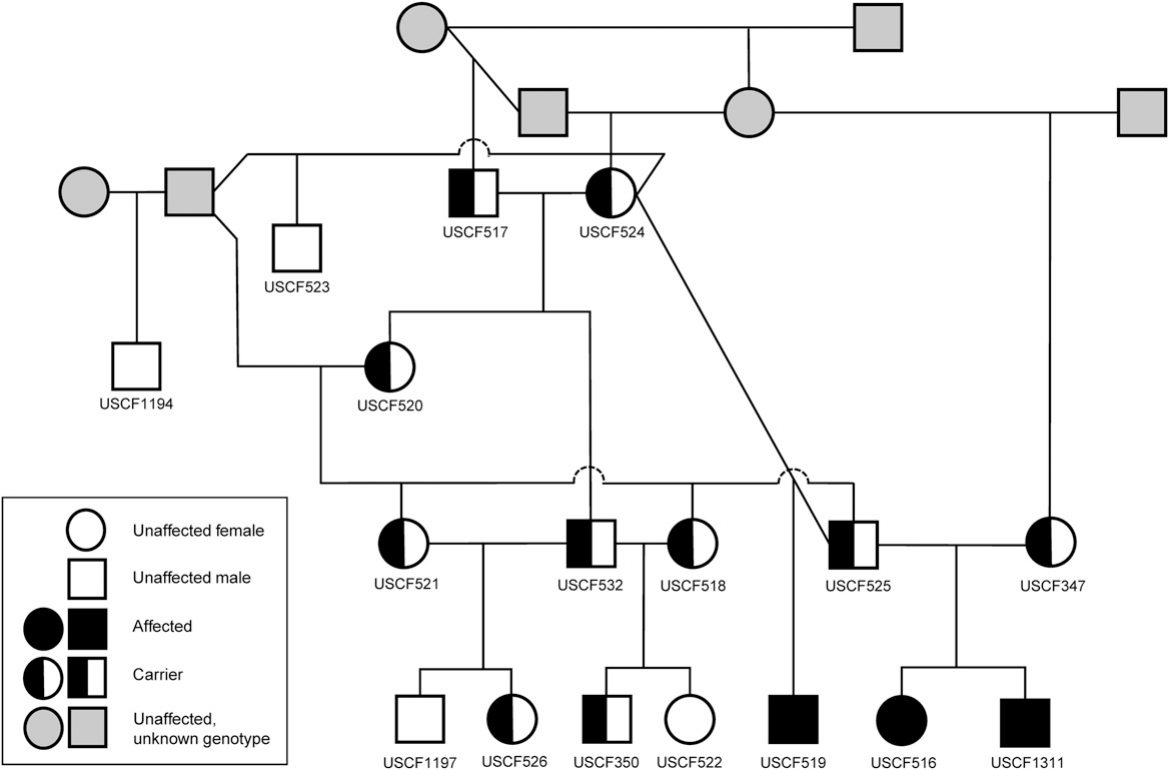
Segregation of the *BBS4* SNP (c.58A > T, p.Lys20*) in the Hungarian Puli family. DNA samples were available for all individuals with an identifier (*n* = 17). PRA is consistent with an autosomal recessive form in this family. Genotypes confirmed through Sanger sequencing represented by unfilled (homozygous wild type *A*/*A*), filled (homozygous mutant *T*/*T*), or half filled (heterozygous *A*/*T*) circles (females) or squares (males) support this mode of inheritance.

**Figure 4:**
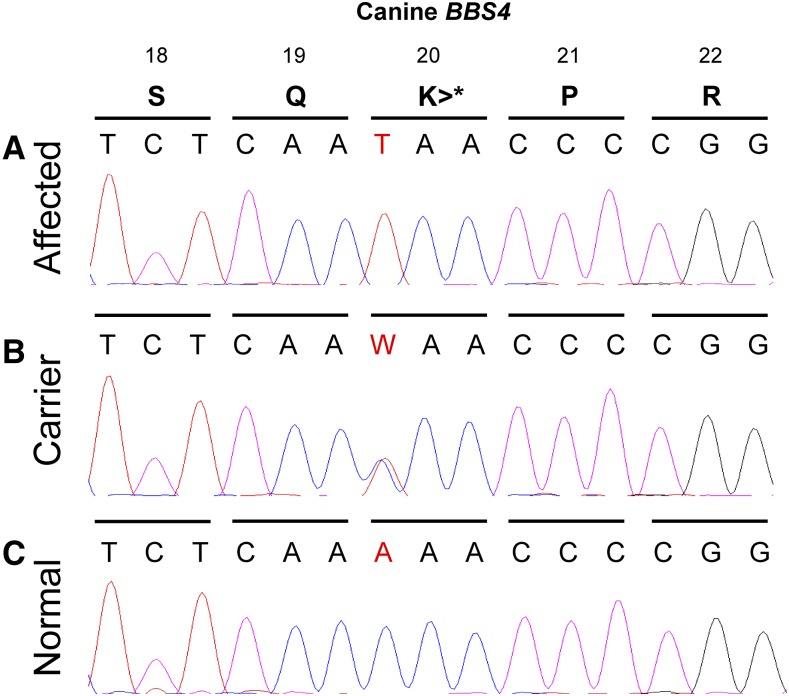
Sanger sequencing of a PCR fragment containing the c.58A > T SNP at position chr30: 36,063,748 on CanFam 3.1 in exon 2 of *BBS4*. The numbers above the amino acid code (S = serine, Q = glycine, K = lysine, P = proline, R = arginine) denote their position in the protein sequence. Nucleotides (W = A or T) are arranged in the 5′ to 3′ direction. (A) All affected Hungarian Puli cases (USCF516, USCF519, and USCF1311) have the *T*/*T* genotype which results in a nonsense mutation (p.Lys20*). (B) Carrier individuals including all parents (USCF347, USCF524, and USCF525) have the *A*/*T* genotype. (C) Unaffected individuals have the *A*/*A* genotype. This is identical to the canine reference sequence (CanFam 3.1 build).

The 103 Hungarian Puli included the three affected animals and 14 others with normal vision from the same kennel (Figure 3). Pedigree relationships for the 14 individuals for which genotyping array data were available were confirmed through identity-by-descent estimations (Table S2). Through Sanger sequencing, we observed that all three affected dogs (USCF516, USCF519, and USCF1311) were homozygous for the variant allele (*T*/*T*), all three obligate carrier parents were heterozygous (*A*/*T*), and the remaining unaffected Hungarian Puli were either heterozygous or homozygous for the wild-type allele (*A*/*A*, Figure 4). All Hungarian Pumi were homozygous for the wild-type allele. Genotypes for each individual in the study can be found in File S1.

An association of *P_CHISQ_* = 3.425*e*^−14^ between the c.58A > T SNP in *BBS4* to the disease phenotype was found for all validated Hungarian Puli genotypes (*n* = 103). When including validated Hungarian Pumi genotypes, the association is *P_CHISQ_* = 3.252*e*^−34^ (*n* = 255). The genotypes are perfectly consistent with an autosomal recessive pattern of inheritance for the 17 Hungarian Puli individuals with pedigree information, which supports the expected segregation pattern for PRA in this breed (Figure 3).

### Assessment of Bbs4^−/−^ mouse phenotypes in the dog

The intact affected male Hungarian Puli (*n* = 1) was found to be subfertile. Semen analysis indicated normal sperm concentration but a low total sperm count (13.65*e*^6^). A high proportion (78%) of sperm had abnormal morphology, predominantly as a consequence of spermatozoa tail defects (74%, Table 2). There were <5% of sperm with normal motility.

**Table 2:** Semen analysis report of affected Hungarian Puli

	Normal Dog (Schaer 2009)	Affected Hungarian Puli
Normal morphology (%)	≥80	22
Abnormal morphology (%)	—	—
Head defects	—	18
Midpiece defects	—	2
Tail defects	—	74
Normal motility (%)	≥70	<5
Concentration (number per ml)	4–400*e*^6^	6.5*e*^6^
Total sperm	100–3000*e*^6^	13.65*e*^6^
Volume (ml)	0.4–40	2.1
Color	Cloudy white	Transparent

Normal canine semen characteristics were obtained from Schaer 2009.

## Discussion

In this study, we identify a putative functional variant that is highly associated with the PRA disease phenotype in the Hungarian Puli breed. The variant occurs in a novel canine PRA gene. A preliminary study suggested that it was likely that a novel gene was causing disease in this family because no obvious functional variants were identified in the exons or promoters of any of 53 previously described PRA genes (Chew *et al.* 2017). Using genotyping array data and WGS data of a parent-proband trio (USCF525, USCF347, and USCF516) and an additional half sibling case (USCF519), we identify a nonsense SNP (p.Lys20*) in exon 2 of *BBS4* that is significantly associated with disease (*P_CHISQ_* = 3.425*e*^−14^, *n* = 103). The associated SNP perfectly segregates in an autosomal recessive mode of inheritance. The mutation results in truncation at the N-terminal of the translated BBS4 protein, reducing a 520 amino acid protein down to a 19 amino acid peptide. We predict that nonsense-mediated decay of *BBS4* messenger RNA would hinder the expression of functional BBS4 protein (Popp and Maquat 2013). In humans and mice, *BBS4* is associated with the syndromic disease, BBS. We also provide some evidence that this form of PRA in the dog is part of a syndromic disease. There are now two BBS genes implied in canine PRA (*BBS4* and *TTC8*; Downs *et al.* 2014b). As BBS has not been previously reported in the dog, future PRA cases should be monitored for BBS phenotypes and gene mutations as they may provide a potential canine model for human disease.

*BBS4* is one of eight evolutionarily conserved proteins that together form a multi-protein complex referred to as the BBSome (Nachury *et al.* 2007; Loktev *et al.* 2008). This complex localizes to primary cilia, a small hair-like organelle that is present on almost all vertebrate cells. Cilia play a vital role in many developmental pathways that occur during vertebrate embryogenesis enabling correct organ differentiation and spatial organization within the body. Primary cilia mediate multiple cell signaling activities in nondividing cells, responding to both mechanical and chemosensory stimuli in multiple body systems as they contain tissue specific sensory receptors (Singla and Reiter 2006; Goetz and Anderson 2010). The ubiquity of primary cilia and the concurrent differences in characteristics that they possess depending on their residing cell type give ciliopathies their clinical heterogeneity. Presumably, a dysfunctional protein that normally localizes to cilia of only one cell type will result in nonsyndromic disease, while proteins essential to cilia on multiple cell types such as those involved in its maintenance will result in syndromic disease.

The formation and maintenance of cilia are highly dependent on the bidirectional (anterograde and retrograde) movement of nonmembrane-bound particles between the cell body and the tip of the cilia via its axonemal microtubules. The mechanism for this is referred to as intraflagellar transport (IFT) (Rosenbaum and Witman 2002). While the BBSome is not directly required for cilia formation, it is essential for the trafficking and organization of IFT complexes and hence has an indirect role in ciliary maintenance (Wei *et al.* 2012). Disruption in any of the BBSome genes (among 11 others that are not part of the BBSome) can cause failure of this mechanism, resulting in the rare ciliopathy, BBS (Suspitsin and Imyanitov 2016). The degree of importance of each BBS protein and their effect on the ability for the BBSome to carry out its ciliary functions within the various cell types remains elusive.

Studies of human BBS type 4 (OMIM #615982) and *Bbs4*-null mice show that functions of the canine BBS4 protein are consistent with these theories. Structurally normal primary and motile cilia were observed in knockout mice, suggesting that *BBS4* is not required for the formation of cilia (Mykytyn *et al.* 2004). All affected individuals including the dogs used in this study experience retinal degeneration, despite having normal vision at a very young age (Iannaccone *et al.* 1999, 2005; Riise *et al.* 2002; Mykytyn *et al.* 2004; Li *et al.* 2014). This suggests that cilia are correctly formed, however IFT of newly synthesized proteins in the inner segment to the outer segment of photoreceptor cells is compromised, as the only route between the two is through connecting cilia (Marszalek *et al.* 2000; Mykytyn *et al.* 2004). These proteins are essential to photoreceptor maintenance and without these, the photoreceptor cells undergo apoptosis.

BBS is recognized as a syndromic disease, however in the dog, the disease may appear as nonsyndromic PRA. Like canine PRA, BBS is typically inherited in an autosomal recessive manner, except for one report of triallelic inheritance **(**Katsanis *et al.* 2001; Forsythe and Beales 2013). In human BBS type 4, symptoms that are observed in addition to RP include obesity, hypogenitalism, polydactyly, mental retardation, renal anomalies, and decreased olfaction (Iannaccone *et al.* 1999, 2005; Riise *et al.* 2002; Li *et al.* 2014; Aksanov *et al.* 2014). The severity and frequency of occurrence of each of these symptoms is variable like for all types of BBS, and clinical diagnosis is based on the presence of three to four primary and two secondary symptoms (Forsythe and Beales 2013). The difference in the underlying genetic mutation for reports of BBS type 4 is likely to contribute to this heterogeneity.

The affected Hungarian Puli in this study were predicted to have no functional BBS4, so we compared their phenotypes to those observed in *Bbs4*-null mice. In these mice, obesity and a complete lack of spermatozoa flagella were observed in addition to retinal degeneration (Mykytyn *et al.* 2004). In the dog, we observed all of these phenotypes but found that canine spermatozoa flagella were not as severely affected as those in the mouse. We observed 22% of sperm with normal morphology in the dog; however, a large proportion of abnormal sperm had defective flagella (74%) and a very small proportion were motile (<5%; Table 2). This suggests that *BBS4* is only of moderate importance to flagella formation but is necessary for providing motility in the dog. More canine samples are required to confirm this.

The difficulty with differentiating nonsyndromic and syndromic disease in companion animals such as the dog is that many of the concurrent symptoms may not be diagnosed or recognized. Obesity is common with 26–43% of pure- and mixed-breed dogs classed as overweight in an Australian survey (McGreevy *et al.* 2005). As it is widely recognized as a nutritional disease, many people would underestimate the genetic component of this phenotype. Further, in Australia many companion animals are desexed prior to maturity, limiting the opportunity to recognize fertility deficits. Other symptoms such as learning or developmental delay and decreased olfaction may be difficult to assess in animals. For these reasons, we recommend that all human BBS genes might be considered as potential candidate genes for cases of canine PRA with unknown genetic causation. Further studies are required to confirm that *BBS4* causes syndromic disease in the dog and this should be monitored as it may potentially be a useful large animal model for human BBS.

## Supplementary Material

Supplemental material is available online at www.g3journal.org/lookup/suppl/doi:10.1534/g3.117.043109/-/DC1.
