# Supplementary material for: A Coding Variant in the Gene Bardet-Biedl Syndrome 4 (BBS4) Is Associated with a Novel Form of Canine Progressive Retinal Atrophy
Source: G3 (Bethesda). 2017 May 22;7(7):2327–35. doi: 10.1534/g3.117.043109 (PMC5499139; doi:10.1534/g3.117.043109)
Supplement: Supplementary file 2 [file 2327FileS2.pdf]

File S2. Completion of the current *BBS4* annotation in CanFam3.1 reference genome

Introduction

Exon 1 of BBS4 is not annotated in the most current reference sequence (CanFam3.1). Its absence is evident by a lack of an initiation codon. When observing a multiple sequence alignment of BBS4 protein sequences from a variety of vertebral species including human, orangutan, mouse, rat, cow, cat and elephant it is clear that the dog is lacking the first part of the transcript. We hypothesized that exon 1 resided in a reference genome assembly gap ~9.7 Kb upstream to the current BBS4 annotation and adjacent to a region of high guanine-cytosine density.

Methods

Using the popular *de novo* aligner Velvet version 1.2.10 (Zerbino and Birney 2008), we attempted to resolve this gap by assembling unmapped reads and reads that partially mapped to the vicinity of the gap in chromosome 30 from four Hungarian Puli dogs. The initial attempt at assembly was unsuccessful in building a contig that completely resolved the gap. Alternatively, we performed a manual alignment using sequences from unmapped mates of reads that had aligned adjacent to the gap. A multiple sequence alignment with the assembled contig and exon 1 of human (NR_033028.4), mouse (NM_175325.3) and cat (XM_011282956.1) was performed using Clustal Omega (Sievers *et al.* 2011). The assembled contig was translated into an amino acid sequence using ExPASy's translate tool (Gasteiger *et al.* 2003). We similarly aligned the predicted canine BBS4 protein corresponding to exon 1 to human (NP_149017.2), mouse (NP_780534.1) and cat (XP_011281258.1) BBS4 proteins.

Results and Conclusions

Multiple sequence alignment of the contig produced from manual assembly revealed that putative exon 1 of *BBS4* in the dog is identical to that of the domestic cat (*Felis catus*) and differs from the human sequence by two nucleotides (Figure S1). Thus, the complete BBS4 protein in dogs consists of 520 amino acids encoded by 1,560 base pairs of mRNA organised into 16 exons on chromosome 30 of CanFam 3.1. Protein sequences corresponding to exon 1 of *BBS4* are identical for dog and cat but differ to human and mouse by one and five amino acids respectively (Figure S2).

```
Canine Contig   AGCCAAGATGGCTGAGGAGAGGCTGGCGACGGTGAGCGCCGACCTGCCGCTCGGTGTCCC
Homo sapiens    -------ATGGCTGAGGAGAGAGTCGCGACG-----------------------------
Mus musculus    -------ATGGCTGAAGTGAAGCTTGGGATG-----------------------------
Felis catus     -------ATGGCTGAGGAGAGACTCGCGACG-----------------------------
                       ******** * **   * * ** *
```

Figure S1. Multiple sequence alignment of a manually assembled canine contig with exon 1 of *BBS4* of human (*Homo sapiens*), mouse (*Mus musculus*) and domestic cat (*Felis catus*) nucleotide sequences. The canine contig was assembled using reads from four Hungarian Puli dogs. Reads include unmapped mates of pairs that aligned adjacent to a reference genome gap

on chromosome 30, putative to the location of *BBS4* exon 1. An asterisk denotes full identity of the nucleotide across species.

```
Canis familiaris      MAEERLATRTQFPASAESQKPRLKK
Felis catus           MAEERLATRTQLPASAESQKPRLKK
Homo sapien           MAEERVATRTQFPVSTESQKPRQKK
Mus musculus          MAEVKLGMKTQVPASVESQKPRSKK
                      *** ::. :**.*.*.****** **
```

Figure S2. Multiple amino acid sequence alignment of partial canine BBS4 protein with domestic cat (*Felis catus*), human (*Homo sapien*) and mouse (*Mus musculus*) homologs corresponding to exon 1 and 2 only. The canine protein sequence corresponding to exon 1 (highlighted in grey) and exon 2 was obtained from translation of genomic sequence of a contig produced by manual *de novo* assembly of canine Illumina HiSeq 2000 reads.

The complete mRNA and amino acid sequences for canine *BBS4* have been deposited in Genbank (KX290494).

Literature Cited

Gasteiger, E., A. Gattiker, C. Hoogland, I. Ivanyi, R. D. Appel *et al.*, 2003 ExPASy: The proteomics server for in-depth protein knowledge and analysis. Nucleic Acids Res. 31(13): 3784–8.

Sievers, F., A. Wilm, D. Dineen, T. J. Gibson, K. Karplus *et al.*, 2011 Fast, scalable generation of high-quality protein multiple sequence alignments using Clustal Omega. Mol. Syst. Biol. 7: 539.

Zerbino, D. R., and E. Birney, 2008 Velvet: algorithms for de novo short read assembly using de Bruijn graphs. Genome Res. 18(2): 821–9.
