# Supplementary material for: A Coding Variant in the Gene Bardet-Biedl Syndrome 4 (BBS4) Is Associated with a Novel Form of Canine Progressive Retinal Atrophy
Source: G3 (Bethesda). 2017 May 22;7(7):2327–35. doi: 10.1534/g3.117.043109 (PMC5499139; doi:10.1534/g3.117.043109)
Supplement: Supplementary file 3 [file 2327TableS1.pdf]

Table S1. Candidate genes for progressive retinal atrophy in the Hungarian Puli dog breed. Candidates were selected from regions with a highest density of SNP markers (chromosome 30, 25.3 – 40.0 Mb; chromosome 4, position 0.5 – 10.5 Mb; and chromosome 20, 9.5 – 20.2 Mb on CanFam 3.1) that were concordant to a recessive pattern of inheritance. All genes have a phenotypic connection to vision as indicated by the Mouse Genome Browser.

Gene	CanFam 3.1 Position	Ensembl Transcript ID
SPRTN	chr4: 8,120,974-8,133,016	ENSCAFT00000018713
GNPAT	chr4: 8,152,743-8,190,051	ENSCAFT00000018796
ACTA1	chr4: 9,812,782-9,815,574	ENSCAFT00000013094
ITPR1	chr20: 12,747,751-13,065,329	ENSCAFT00000009530
PDZRN3	chr20: 18,838,303-18,861,714	ENSCAFT00000043571
RYBP	chr20: 19,654,014-19,729,510	ENSCAFT00000010397
CPLX3	chr30: 37,888,801-37,892,561	ENSCAFT00000028507
CSK	chr30: 37,866,652-37,869,289	ENSCAFT00000028485
STRA6	chr30: 37,332,568-37,346,312	ENSCAFT00000048873
BBS4	chr30: 36,063,713-36,109,202	ENSCAFT00000028102
HEXA	chr30: 35,838,158-35,843,722	ENSCAFT00000028088
GLCE	chr30: 33,142,327-33,208,130	ENSCAFT00000046216
CLN6	chr30: 32,246,411-32,264,240	ENSCAFT00000027690
SMAD3	chr30: 31,246,313-31,360,098	ENSCAFT00000027577
MAP2K1	chr30: 30,683,192-30,760,479	ENSCAFT00000043934
MEGF11	chr30: 30,234,191-30,446,063	ENSCAFT00000027347
SLC24A1	chr30: 29,967,634-29,996,958	ENSCAFT00000027314
RAB8B	chr30: 27,784,338-27,845,901	ENSCAFT00000026890

**NR2E3 (*chr30: 35,378,421-35,381,822) was excluded as it is a known canine PRA gene. A preliminary study (Chew et al., 2017 [Animal Genetics in press]) confirms that no putative variants are present in this gene.

Literature Cited

Chew, T., B. Haase, C. E. Willet, and C. M. Wade, 2017 Exclusion of known progressive retinal atrophy genes for blindness in the Hungarian Puli. Anim Genet. In press.
