# Supplementary material for: A Coding Variant in the Gene Bardet-Biedl Syndrome 4 (BBS4) Is Associated with a Novel Form of Canine Progressive Retinal Atrophy
Source: G3 (Bethesda). 2017 May 22;7(7):2327–35. doi: 10.1534/g3.117.043109 (PMC5499139; doi:10.1534/g3.117.043109)
Supplement: Supplementary file 4 [file 2327TableS2.pdf]

**Table S2.** Relationships between 14 Hungarian Puli individuals from the same pedigree estimated through proportion of identity by descent (IBD) calculations performed using PLINK (Purcell *et al.* 2007). Relationships were obtained from pedigree records (Australian National Kennel Council).

| Individual 1 | Individual 2 | Relationship | Proportion IBD |
|---|---|---|---|
| USCF532 | USCF347 | OT | 0 |
| USCF532 | USCF350 | PO | 0.5 |
| USCF532 | USCF516 | OT | 0 |
| USCF532 | USCF517 | PO | 0.5 |
| USCF532 | USCF518 | HS | 0.244 |
| USCF532 | USCF519 | HS | 0.1755 |
| USCF532 | USCF520 | FS | 0.3737 |
| USCF532 | USCF522 | PO | 0.5 |
| USCF532 | USCF523 | HS | 0.2037 |
| USCF532 | USCF524 | PO | 0.5 |
| USCF532 | USCF525 | OT | 0.1626 |
| USCF532 | USCF526 | PO | 0.5 |
| USCF532 | USCF521 | OT | 0.2215 |
| USCF347 | USCF350 | OT | 0 |
| USCF347 | USCF516 | PO | 0.5 |
| USCF347 | USCF517 | OT | 0.0551 |
| USCF347 | USCF518 | OT | 0 |
| USCF347 | USCF519 | OT | 0.0577 |
| USCF347 | USCF520 | OT | 0 |
| USCF347 | USCF522 | OT | 0 |
| USCF347 | USCF523 | OT | 0.1117 |
| USCF347 | USCF524 | HS | 0.2835 |
| USCF347 | USCF525 | OT | 0 |
| USCF347 | USCF526 | OT | 0 |
| USCF347 | USCF521 | OT | 0 |
| USCF350 | USCF516 | OT | 0.0656 |
| USCF350 | USCF517 | OT | 0.3262 |
| USCF350 | USCF518 | PO | 0.5 |
| USCF350 | USCF519 | OT | 0.1257 |
| USCF350 | USCF520 | OT | 0.3004 |
| USCF350 | USCF522 | FS | 0.4293 |
| USCF350 | USCF523 | OT | 0.2823 |
| USCF350 | USCF524 | OT | 0.393 |
| USCF350 | USCF525 | OT | 0.2628 |
| USCF350 | USCF526 | HS | 0.2777 |
| USCF350 | USCF521 | OT | 0.2286 |
| USCF516 | USCF517 | OT | 0.183 |

| | | | |
|---|---|---|---|
| USCF516 | USCF518 | OT | 0.1856 |
| USCF516 | USCF519 | HS | 0.3014 |
| USCF516 | USCF520 | OT | 0.1565 |
| USCF516 | USCF522 | OT | 0 |
| USCF516 | USCF523 | OT | 0.1211 |
| USCF516 | USCF524 | OT | 0.2613 |
| USCF516 | USCF525 | PO | 0.5 |
| USCF516 | USCF526 | OT | 0.1235 |
| USCF516 | USCF521 | OT | 0.2273 |
| USCF517 | USCF518 | OT | 0.5 |
| USCF517 | USCF519 | OT | 0.2414 |
| USCF517 | USCF520 | PO | 0.5037 |
| USCF517 | USCF522 | OT | 0.3605 |
| USCF517 | USCF523 | OT | 0.2289 |
| USCF517 | USCF524 | HS | 0.3298 |
| USCF517 | USCF525 | OT | 0.3651 |
| USCF517 | USCF526 | OT | 0.3112 |
| USCF517 | USCF521 | OT | 0.3831 |
| USCF518 | USCF519 | OT | 0.2845 |
| USCF518 | USCF520 | PO | 0.5 |
| USCF518 | USCF522 | PO | 0.5 |
| USCF518 | USCF523 | HS | 0.374 |
| USCF518 | USCF524 | OT | 0.5 |
| USCF518 | USCF525 | OT | 0.3875 |
| USCF518 | USCF526 | OT | 0.3268 |
| USCF518 | USCF521 | FS | 0.5548 |
| USCF519 | USCF520 | HS | 0.3208 |
| USCF519 | USCF522 | OT | 0 |
| USCF519 | USCF523 | HS | 0.2537 |
| USCF519 | USCF524 | PO | 0.5 |
| USCF519 | USCF525 | PO | 0.5 |
| USCF519 | USCF526 | OT | 0.1335 |
| USCF519 | USCF521 | HS | 0.3223 |
| USCF520 | USCF522 | OT | 0.3634 |
| USCF520 | USCF523 | HS | 0.2515 |
| USCF520 | USCF524 | PO | 0.5049 |
| USCF520 | USCF525 | PO | 0.5 |
| USCF520 | USCF526 | OT | 0.403 |
| USCF520 | USCF521 | PO | 0.5 |
| USCF522 | USCF523 | OT | 0.2233 |
| USCF522 | USCF524 | OT | 0.3924 |
| USCF522 | USCF525 | OT | 0.2033 |
| USCF522 | USCF526 | HS | 0.2459 |

| | | | |
|---|---|---|---|
| USCF522 | USCF521 | OT | 0.2668 |
| USCF523 | USCF524 | PO | 0.5 |
| USCF523 | USCF525 | OT | 0.2419 |
| USCF523 | USCF526 | OT | 0.1263 |
| USCF523 | USCF521 | HS | 0.3722 |
| USCF524 | USCF525 | OT | 0.3249 |
| USCF524 | USCF526 | OT | 0.3451 |
| USCF524 | USCF521 | OT | 0.5 |
| USCF525 | USCF526 | OT | 0.2616 |
| USCF525 | USCF521 | FS | 0.4689 |
| USCF526 | USCF521 | PO | 0.5 |

Samples were genotyped on the CanineHD BeadChip array. Relationships provided by pedigree records are consistent with proportion of IBD estimations that were expected depending on the type of relationship. Parent-offspring (PO) relationships have an expected IBD = 0.5; full-sibling (FS) relationships have an expected IBD = 0.5; half-sibling relationships have an expected IBD = 0.25. OT indicates ‘other’ relationships.

**References**

Purcell, S., B. Neale, K. Todd-Brown, L. Thomas, M. A. R. Ferreira *et al.*, 2007 PLINK: a tool set for whole-genome association and population-based linkage analyses. Am. J. Hum. Genet. 81(3): 559–75.
